# The Roles of Dietary PPAR**γ** Ligands for Metastasis in Colorectal Cancer

**DOI:** 10.1155/2008/529720

**Published:** 2008-06-05

**Authors:** Hiroki Kuniyasu

**Affiliations:** Department of Molecular Pathology, School of Medicine, Nara Medical University, 840 Shijo-cho, Kashihara, Nara 634-8521, Japan

## Abstract

Dietary peroxisome proliferator-activated receptor (PPAR)*γ* ligands, linoleic acid (LA) and conjugated linoleic acid (CLA), showed anticancer effects in colorectal carcinoma cells. LA is metabolized by two pathways. Cyclooxygenase (COX)-2 produces procarcinogenic prostaglandin E2, whereas 15-lipoxygenase (LOX)-1 produces PPAR*γ* ligands. The 15LOX-1 pathway, which is dominant in colorectal adenomas, was downregulated and inversely COX-2 was upregulated in colorectal cancer. LA and CLA inhibited peritoneal metastasis of colorectal cancer cells in nude mice. The inhibitory effect was abrogated by PPAR*γ* antisense treatment. A continuous LA treatment provided cancer cells quiescence. These quiescent cells formed dormant nests in nude mice administrated LA. The quiescent and dormant cells showed downregulated PPAR*γ* and upregulated nucleostemin. Thus, short-term exposure to dietary PPAR*γ* ligands inhibits cancer metastasis, whereas consistent exposure to LA provides quiescent/dormant status with possible induction of cancer stem and/or progenitor phenotype. The complicated roles of dietary PPAR*γ* ligands are needed to examine further.

## 1. INTRODUCTION

Colorectal cancer is the third most common malignant neoplasm worldwide and the third leading cause of cancer deaths in Japan [1]. The frequency of colorectal cancer in Japan doubled in the last three decades according to the alteration of life style from Japanese to Western [2]. Especially, the increase in fat intake and decrease in fiber intake have been regarded as the most important nutritional influence on colon cancer development [3, 4]. In this review, we focused on the fatty acids, which possess ligand activity for peroxisome proliferator-activated receptor (PPAR)*γ* in foods. PPAR*γ* is activated by endogenous secreted prostaglandins and fatty acids. 15-deoxy-*δ*(12,14)-prostaglandin J2 is a strong endogenous ligand of PPAR*γ* [5]. Linoleic acid (LA) is one of the essential fatty acids, which we must intake from food. Metabolic products of LA, such as 9-hydroxyoctadecadienoic acid (9-HODE), 13-hydroxyoctadecadienoic acid (13-HODE), and 13-oxooctadecadienoic acid (13-OXO), are known as PPAR*γ* ligands [6]. Conjugated linoleic acid is a stereoisomer of LA [7]. CLA is contained in beef, lamb, and also in vegetable oils [8]. Roles of these dietary PPAR*γ* ligands on PPAR*γ* activation are not still unclear. Colorectal cancer is a good model for influence of nutritional factors to cancer development and progression [9, 10]. In this review, roles of LA and CLA for colorectal cancer progression and therapeutic possibility are discussed as dietary PPAR*γ* ligands.

## 2. METABOLIC PATHWAYS OF LINOLEIC ACID

Prostaglandins (PGs) are bioactive lipids derived from the metabolites of membrane polyunsaturated fatty acids (PUFAs), and play important roles in a number of biological processes [11]. Cyclooxygenases-2 (COX-2)-dependent overproduction of PGE2 is hypothesized to be an important part of sustained proliferative and chronic inflammatory conditions [12, 13]. Several in vivo studies hypothesize that a high amount of *ω*-6 PUFA such as LA might enhance colorectal carcinogenesis via stimulation of colonic epithelial cell proliferation [14–17]. In fact, rats treated with a genotoxic agent, azoxymethane (AOM), and fed a diet supplemented with LA develop more tumors than those treated with AOM alone [18].

The oxidative metabolites of LA, in particular, 9-HODE, 13-HODE, and 13-OXO, have biological effects as a PPAR*γ* ligand [6]. CLA, a strong ligand for PPAR*γ*, has a substantial anticarcinogenic effect [8, 19]. Synthesized PPAR*γ* ligands including troglitazon have been shown to be effective chemopreventive agents in a rat model of carcinogenesis and in AOM-induced colon cancer in mice [20]. If LA provides PPAR*γ* ligands, LA might act as an anticarcinogenic agent.

Arachidonic acid is a fatty acid and a component of lipid membranes, and also a major substrate for lipoxygenase enzymes. 15lipooxygenase-1 (15LOX-1) is known for its anti-inflammatory properties and has a profound influence on the development and progression of cancers [21, 22]. Recent studies show that ligand activation of PPAR*γ* in colorectal cancer cells attenuates colonic inflammation and causes a reduction growth via the induction of apoptosis [23, 24]. Furthermore, it has been reported that a number of metabolites generated by 15LOX-1 can function as endogenous activators and ligands for PPAR*γ* [25, 26]. We confirmed the anticarcinogenic effect of LA by in vitro transformation assay using a rat intestinal cell line, IEC6, which expressed 15LOX-1, but not COX2. LA treatment inhibited AOM-induced transformation in IEC6 cells [27]. Many literatures reported that PPAR*γ* possesses an anticarcinogenic effect in colorectal cancer [20]. Moreover, a decrease in PPAR*γ* expression is associated with cancer metastasis [28, 29]. Inhibitory effect of PPAR*γ* to cancer metastasis is reported in several cancers, such as nonsmall cell lung cancer, colon cancer, thyroid cancer, and breast cancer [30–33].

## 3. SWITCHING OF LA METABOLIC PATHWAYS FROM 15LOX-1 TO COX-2 IN COLORECTAL CANCER DEVELOPMENT

Inhibitory effect of LA on intestinal epithelial cell transformation elucidated above suggests that 15LOX-1 LA metabolism suppresses colon carcinogensis [27]. We next focused on the dual roles of LA in human colon cancer development. Expression of 15LOX-1 and COX-2 was examined in human colon adenoma and carcinoma to elucidate the balance of the two LA metabolic pathways in malignant transformation of human colon epithelium.

We examined the expressions of COX-2 and 15LOX-1 in 54 adenomas, 21 carcinoma-in-adenoma lesions, and 36 serosa-invading advanced carcinomas in the colon [34]. We examined 15LOX-1 mRNA and COX-2 protein by in situ hybridization and immunohistochemistry, respectively. In the nonpathological colon mucosa, which expressed 15LOX-1 but not COX-2, proliferation of colon epithelial cells was controlled at constitutive levels. 15LOX-1 mRNA was found in 96% of adenomas, 43% of adenoma in carcinoma-in-adenoma lesions, and 10% of carcinoma in carcinoma-in-adenoma lesions, but not in advanced carcinoma (*P* < .0001). In contrast, COX-2 production was found in 11% of adenomas, 52% of adenoma in carcinoma-in-adenoma lesions, 71% of carcinoma in carcinoma-in-adenoma lesions, and 92% of advanced carcinoma (*P* < .0001). Concurrence of COX-2 induction with 15LOX-1 downregulation was found in 6% adenomas, in 33% adenoma components, and 71% carcinoma components of carcinoma-in-adenoma lesions (all mucosal cancer), in 89% cases in nonmetastatic serosa-invading carcinomas, and in 100% cases of nodal metastasized carcinomas. Our data showed that induction of COX-2 expression and downregulation of 15LOX-1 were sequentially increased from adenomas, adenoma components, and carcinoma components in carcinoma-in-adenoma lesions, to invasive carcinomas. Interestingly, low grade-adenoma components with in carcinoma-in-adenoma lesions showed COX-2 expression and 15LOX-1 downregulation at more frequency than low grade-adenomas, which suggests that the biological property is different in the same histological atypia. In contrast, high grade-adenoma components showed no difference from high grade-adenomas in expressions of COX-2 and 15LOX-1, which suggests that high grade-adenomas might already possess malignant potential as high as adenoma components in carcinoma-in-adenoma lesions. This sequential alteration of concurrence of COX-2 induction with 15LOX-1 downregulation possibly shows close association of the switching of LA-metabolizing pathways with colon cancer development and progression.

15LOX-1 is revealed as an apoptosis inducer in human cancers and inhibits cancer progression. The reduction of 15LOX-1 and the isoform 15LOX-2 is correlated with the disease progression of breast cancer and the poor clinical outcome [35]. Induction of 15LOX-1 provides apoptosis in oral cancer [36]. 15LOX-1 expression is downregulated in colon adenomas, and ectopic expression of 15LOX-1 induces apoptosis in Caco-2 colon cancer cells [37].

In expression of 15LOX-1, inflammatory cytokines play an important role. Interleukin (IL)-4 and IL-13 induce 15LOX-1 expression via Jak2/Tyk2/Stats pathway [36, 38–40]. In prostate cancer, ratio of *ω*-3/*ω*-6 fatty acids is associated with expressions of 15LOX-1 and COX-2 [41]. 15LOX-1 induction by nonsteroidal anti-inflammatory drugs (NSAIDs), such as sulindac sulfone, provides apoptosis in SW480 colon cancer cells. Sulindac sulfone inhibits GMP (cGMP)-phosphodiesterases and activates protein kinase G, which enhances 15LOX-1 expression transcriptionally [42]. GATA-6 transcriptional factor is involved in NSAID-induced 15LOX-1 induction in RKO and DLD-1 colon cancer cells [43]. GATA-6 activates 15LOX-1 promoter in Caco-2 colon cancer cells but not in the cells induced differentiation by sodium-butyrate [44]. These are supported by conventional NSAIDs activates PPAR*γ* [45]. COX-2 expression also involves Jak2/Stats pathway and nuclear factor (NF*κ*B) [46–48]. Activation of PPAR*γ* downregulates COX-2 expression by inhibition of NF*κ*B and activator protein (AP)-1 [49]. This negative regulation of COX-2 expression by PPAR*γ* activation might be one of the mechanisms of reversal expressions between 15LOX-1 and COX-2. Furthermore, promoter DNA methylation is responsible for silencing 15LOX-1 expression, which is reversed by 5-aza-2-deoxycytidine treatment [50, 51]. The epigenetic alteration might be a trigger to switch 15LOX-1 repression and COX-2 upregulation along with malignant transformation and disease progression in colorectal cancer. Thus, switching of LA metabolism from 15LOX-1 to COX-2 is thought to be a common mechanism to escape from antitumoral effect of LA.

## 4. ANTITUMOR EFFECT OF CLA

We next argue the effect of CLA, which is an isomer of linoleic acid and is established as a PPAR*γ* ligand without metabolization by 15LOX-1. CLA is a natural content of some foods, such as beef, lamb, and also vegetable oils [8]. CLA is one of essential fatty acids, which possesses characteristic bioactive properties [8]. CLA is composed of positional- and stereoisomers of octadecadienoate (18 : 2). Chemoprotective properties of CLA are reported in experimental cancer models and in vitro examinations [8, 19]. Differently from LA, CLA is not a precursor of prostaglandines. CLA activates PPAR*γ* as a ligand [7, 52]. Through activation of PPAR*γ*, CLA might act as an antimetastatic agent as well as an anticarcinogenic agent. We examined the antimetastatic effect of CLA on peritoneal dissemination [53]. Cell growth of MKN28 human gastric cancer cells and Colo320 human colon cancer cells was suppressed by CLA in a dose-dependent manner with an increment in apoptosis. CLA significantly inhibited invasion into type IV collagen-coated membrane of MKN28 and Colo320 cells. CLA-induced growth inhibition was recovered by the exposure to antisense S-oligodeoxynucleotides (ODN) for PPAR*γ* in both cell lines. BALB/c nu-nu mice were inoculated with MKN28 and Colo320 cells into their peritoneal cavity, and administrated with CLA intraperitoneally (weekly, 4 times). CLA treatment did not affect food intake or weight gain of mice. CLA treatment significantly decreased metastatic foci of both cells in the peritoneal cavity. Survival rate in mice inoculated with MKN28 or Colo320 cells was significantly recovered by CLA treatment. PPAR*γ* initiates transcription of genes associated with energy homeostasis, cell growth, and anti-/proinflammatory effect [24, 54–57]. Protein production in MKN28 and Colo320 cells treated with CLA showed a decrease in epidermal growth factor receptor (EGFR) and transforming growth factor (TGF)-*α* and an increase in Bax. Our results showed that CLA inhibits cell growth and invasion, and induces apoptosis in cancer cells. Our data are supported with the reports, which indicate that PPAR*γ* downregulates EGFR, and upregulates Bax, p21Waf-1, and E-cadherin, which are associated with antiproliferative, proapoptotic, and prodifferentiation effects [33, 58–60].

We also reported the tumor suppressive effect of CLA on established peritoneal tumors using a syngeneic mouse peritoneal metastasis model [61]. C57BL6 mice were inoculated with LL2 cells into their peritoneal cavity. Two weeks after the inoculation, colonized peritoneal cancer foci (2.2 ± 0.4 mm in diameter) were treated with CLA administrated intraperitoneally (600 pmol/mouse, weekly, twice). CLA treatment decreased the number of peritoneal tumors to 26% of that in untreated mice (*P* < .0001). CLA treatment also decreased size of peritoneal tumors to 27% of that in untreated mice (*P* < .0001). In CLA-treated tumors, proliferating cells were decreased (*P* < .0001), whereas apoptotic cells were increased (*P* < .0010). CLA-treated LL2 tumors showed decrease in PPAR*γ* and EGFR proteins, and increase in Bax protein in comparison with untreated tumors.

## 5. ANTITUMOR EFFECT OF LINOLEIC ACID

We confirmed antimetastatic effect of PPAR*γ* by CLA in gastric and colon cancer cells. We next examined antimetastatic effect of LA.

 The effect of LA on peritoneal metastasis was examined using in vitro treatment of cancer cells and mouse peritoneal metastasis models as well as CLA examination. Firstly, cell growth of MKN28 human gastric cancer cells and Colo320 human colon cancer cells were suppressed by LA in a dose-dependent manner with increment of apoptosis. LA significantly inhibited invasion into type IV collagen-coated membrane of MKN28 and Colo320 cells (*P* < .05). The inhibition of growth and invasion and the induction of apoptosis by LA were abrogated by exposure to antisense S-ODN for PPAR*γ*. Moreover, the decrease in 15LOX-1 expression by exposure to antisense S-ODN for 15LOX-1 suppressed the inhibition of growth and invasion and the induction of apoptosis by LA. LA-induced growth inhibition was recovered by the exposure to antisense S-ODN for PPAR*γ* or 15LOX-1. BALB/c nu-nu mice inoculated with MKN28 and Colo320 cells into their peritoneal cavities were administrated IP with LA (weekly, 4 times). The LA treatment significantly diminished the number of metastatic foci of both cells in the peritoneal cavity (*P* < .05). Protein production in MKN28 and Colo320 cells treated with LA showed a decrease in EGFR and an increase in Bax. PPAR*γ* activation is reported to decrease EGFR expression and increase Bax expression [58, 60]. Our data suggest that LA possesses the same mechanism to CLA of PPAR*γ* ligand; however, its efficacy was 10^3^ times weaker than CLA. LA-metabolites thought to be weaker agonists of PPAR*γ*. MKN28 and Colo320 cells expressed both COX-2 and 15LOX-1. At least, relative high concentration of LA might be metabolized dominantly by 15LOX-1, which consequently provides antimetastatic effect in these cells.

Thus, LA and CLA suppress occurrence of cancer metastasis and reduce existing metastatic tumors in animal models. These findings encourage clinical usage of LA and CLA for treatment of cancer metastasis.

## 6. EFFECT OF LA ON CANCER DORMANCY

A short-term treatment with LA or CLA induced apoptosis in a dose-dependent manner through PPAR*γ* activation as described above. On the contrary, in a long-term continuous treatment with adequate concentrations, LA induced reversible cell growth-arrest in cancer cells that escaped from apoptosis [62].

 Cancer cell tumorigenicity in nude mice depends on several factors in cancer cells themselves and their host. To form macroscopical tumors, cancer cells must survive in their host tissue against host immunity, and proliferate with utilization of the host endothelial cells to make tumor vasculature. These steps are similar to those in metastasized cancer cells on the metastasis targets [63]. If cancer cells do not endure the attacks by host immunity, they cannot form tumor. When they survive but not proliferate, the condition resembles quiescent or static dormancy. When they survive and proliferate but do not generate tumor vasculature, the cancer cells stay microscopical cell aggregation; the condition is to be an equivalent condition to tumor dormancy [64].

**Figure 1:**
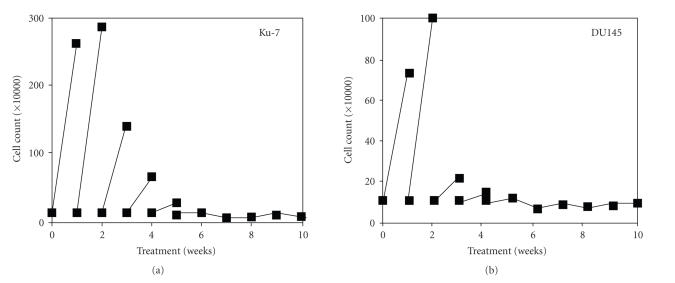
Effect of LA on growth inhibition in Ku-1 bladder cancer cells and DU145 prostate cancer cells by long-term treatment. Ku-7 cells and DU145 cells were continuously treated with LA (100 *μ*g/mL) for the indicated period with weekly reseeding by 1 × 10^5^ cells per well. If cells were less than 1 × 10^5^ cells per well, all cells were reseeded. Standard deviation of each cell number was less than 10% of the value.

In vitro cell growth was suppressed by 48-hour treatment with LA in a dose-dependent manner in MKN28 and Colo320 cells. Continuous treatment with LA induced quiescence in both cells at 5 to 7 weeks after the LA treatment. The finding was also observed in Ku-7 bladder cancer cells and DU145 prostate cancer cells (Figure 1). In LA-induced quiescent cancer cells, protein production of Bcl-2 was increased, whereas Bak, EGFR, and vascular endothelial growth factor (VEGF) levels were decreased [62]. These alterations might be associated with inhibition of cell growth, angiogenesis, and apoptosis [65, 66], which explained well the characteristics of LA-treated cancer cell aggregation. These alterations of protein levels were the same as those in cells treated with PPAR*γ* ligands, toroglitazon, and CLA [62]. The PPAR*γ* expression was also decreased in quiescent MKN28 and Colo320 cells by continuous treatment with LA. The LA metabolites by 15LOX-1 activate mitogen-activated protein kinase (MAPK) and increase PPAR*γ* phosphorylation, but downregulate PPAR*γ* activity [67]. Continuous PPAR*γ* phosphorylation might decrease PPAR*γ* expression by long-term LA treatment.

 When MKN28 and Colo320 cells were inoculated to nude mice subcutaneous tissue, LA-induced quiescent MKN28 and Colo320 cells showed higher tumorigenicity than nontreated cells in nude mice. In the contrary to the tumorigenicity, LA-induced quiescent cancer cells showed 1/10 slower tumor growth than nontreated cells. LA withdrawal after the inoculation provided regrowth in the cancer cells, which subsequently grew into macroscopic tumors. LA-induced quiescent cells were different from growth-arrest cells by aging or senescence, which are irreversible, and refractory to growth factors [68].

In mice treated with LA weekly administration after the inoculation, inoculated quiescent cancer cells did not form macroscopical tumors. Histological examination revealed less than 500 *μ*m-sized cancer-cell aggregations in the inoculation site. These cancer cell nests showed no proliferative activity, no vascularity, and no immune cell infiltration [62]. These features of the cancer cell nests were similar to those in tumor dormancy status [64]. The dormant cells expressed undetectable levels of PPAR*γ* protein, which suggests that inactivation of PPAR*γ* might be associated with tumor dormancy formation. In contrast, withdrawal of LA might break the dormancy status in cancer cells. Moreover, PPAR*γ*-negative dormant cells expressed increased levels of nucleostemin. Nucleostemin possesses a role for maintaining stemness [69, 70]. PPAR*γ* inhibits Wnt and LIF (leukemia inhibitory factor) signaling pathways, which maintain pluripotency and self-renewal of stem cells [5, 71]. Downregulation of PPAR*γ* might induce dedifferentiation and stem cell/progenitor phenotype in cancer cells, which might be associated with cancer dormancy and metastasis.

In this story, several possibilities are considered. Firstly, LA-induced PPAR*γ* downregulation provides stemness in cancer cells. Secondly, LA-induced apoptosis abolishes PPAR*γ*-positive cancer cells and PPAR*γ*-negative cancer stem cell/progenitor cells remain. Thirdly, LA possesses direct effect on cancer stem niche. To confirm the mechanism underlying LA-induced cancer dormancy, further examination is requested to focus on the relation of PPAR*γ* with cancer stem cells.

## 7. CONCLUSION

In this article, we described that the dietary PPAR*γ* ligands, LA and CLA, are deeply involved in colorectal cancer development and progression through PPAR*γ* activation. LA and CLA provide remarkable anticancer effects by short-term treatment. In contrast, long-term continuous treatment with LA induces quiescent and dormancy in cancer cells with PPAR*γ* downregulation. These conditions might be associated with phenotypes of cancer stem cells/progenitor cells. LA is a dietary factor to be taken from food everyday. Cancer cells might be continuously exposed to various concentrations of LA in human body. Taken together, short-term administration of LA and CLA is an effective therapeutic tool for cancer metastasis. We are requested to evaluate the effect of dietary LA on cancer metastasis for prevention of cancer metastasis and cancer dormancy.

## NOMENCLATURE

PPAR: Peroxisome proliferator-activated receptor
LA: Linoleic acid
CLA: Conjugated linoleic acid
COX: Cyclooxygenases
LOX: Lipoxygenase
HODE: Hydroxyoctadecadienoic acid
OXO: Oxooctadecadienoic acid
PG: Prostaglandin
AOM: Azoxymethane
IL: Interleukin
NSAID: Nonsteroidal anti-inflammatory drug
NF: Nuclear factor
AP: Activator protein
ODN: Oligodeoxynucleotides
EGFR: Epidermal growth factor receptor
TGF: Transforming growth factor
VEGF: Vascular endothelial growth factor
MAPK: Mitogen-activated protein kinase
LIF: Leukemia inhibitory factor.
